# Catastrophizing Mediates the Relationship Between the Personal Belief in a Just World and Pain Outcomes Among Chronic Pain Support Group Attendees

**DOI:** 10.1007/s12207-015-9246-y

**Published:** 2016-01-12

**Authors:** Joanna L. McParland, Christina Knussen

**Affiliations:** Department of Psychology, Social Work and Allied Health Sciences, Glasgow Caledonian University, George Moore Building, 70 Cowcaddens Road, Glasgow, G4 OBA UK

**Keywords:** Personal belief in a just world, Chronic pain, Catastrophizing, Distress, Disability

## Abstract

Health-related research suggests the belief in a just world can act as a personal resource that protects against the adverse effects of pain and illness. However, currently, little is known about how this belief, particularly in relation to one’s own life, might influence pain. Consistent with the suggestions of previous research, the present study undertook a secondary data analysis to investigate pain catastrophizing as a mediator of the relationship between the personal just world belief and chronic pain outcomes in a sample of chronic pain support group attendees. Partially supporting the hypotheses, catastrophizing was negatively correlated with the personal just world belief and mediated the relationship between this belief and pain and disability, but not distress. Suggestions for future research and intervention development are made.

## Introduction

Social psychological research suggests that humans have a fundamental aversion to injustice. Instead, they prefer to embrace the justice motive, the need to strive for justice and fairness in life. One key indicator of the justice motive is the belief in a just world: individuals have a need to believe they live in a world in which each person gets what he or she deserves and deserves what he or she gets, whether good or bad (Furnham, 2003). This belief is often described as a positive illusion, but evidence suggests it may reflect the reality of one’s own experience (Sutton et al., 2008). It is theorized to be shaped in childhood under parental and societal influences (Lerner, 1998), and research suggests it fluctuates in strength with cognitive maturity and as a function of, for example, personality and/or demographic variables (Furnham, Swami, Voracek, & Stieger, 2009; Nudelman, 2013). It is proposed that the concern to believe in a just world may be driven by the adaptive function it serves in making the world seem stable, orderly and predictable (see Furnham, 2003). As a result of this, those with a strong just world belief will be often unconsciously motivated to defend their belief when it is threatened by undeserved suffering, the central ingredient of perceived injustice.

A distinction has been made between the belief in a personal just world, in which one usually gets what one deserves, and the belief in a general just world or a belief in a just world for others, in which people in general get what they deserve (Dalbert, 1999). The beliefs have been found to be moderately correlated but theoretically and psychometrically distinct from each other (Dalbert, 1999). Research suggests that the personal just world belief is held more strongly than the general belief (Begue & Bastounis, 2003; Dalbert, 1999; Sutton & Douglas, 2005; Sutton et al., 2008). This may be because it is more fundamental for wellbeing (Alves & Correia, 2010). Consistent evidence suggests that the general just world belief is more predictive than the personal belief of attitudes, while the personal belief is more predictive of indices of psychological wellbeing, including reduced symptoms of stress and depression and increased life satisfaction (Begue & Bastounis, 2003; Dalbert, 1999, 2002; Lipkus, Dalbert, & Siegler, 1996; Sutton & Douglas, 2005; Sutton et al., 2008).

Health and illness research suggests an individual’s belief in justice can affect his or her health outcomes. Theoretically, the experience of illness can threaten or violate the belief in a just world, providing a sense that bad things happen to good people. However, evidence suggests that adopting strategies to maintain or restore the belief in this experience through, for example, taking meaning from it, might address the distress at this violation (Park, Edmondson, Fenster, & Blank, 2008). In this way, the belief in a just world can act as a buffer or personal resource that can prevent or mitigate the potential adverse psychological effects of illness (Dalbert, 2001). A small body of research has found the belief in a just world to be associated with increased happiness among patients with spinal injuries, increased short-term recovery following a myocardial infarction and positive mood among breast cancer sufferers (Agrawal & Dalal, 1993; Bulman & Wortman, 1977; Dalbert & Braun, 1997). These findings suggest that the belief may protect wellbeing at different times during the process of coping with a chronic illness.

More recently, our own research has investigated the role of the belief in a just world as a coping resource among individuals experiencing chronic pain. Chronic pain is defined as pain of any intensity that lasts longer than 3 months (Turk & Okifuji, 2001). The experience of pain by itself does not necessarily drive concerns of injustice or challenge the belief in a just world. Rather, research suggests that issues of social justice and what is deserved are prompted by the experiences people have living their daily life with pain. This may involve how they are treated by others and the struggles they experience attempting to have their medical and financial needs met (McParland, Eccleston, Osborn, & Hezseltine, 2011a). Thus far, our research has found the belief in a just world to confer some benefit in the experience of chronic pain. Our earliest study found that, among a sample of chronic pain support group attendees, the personal just world belief was associated with less pain, disability and distress, while the general just world belief occupied a buffering moderator role. Specifically, the relationship that pain and disability held with psychological distress was attenuated by a stronger general just world belief. Subsequent work only partially replicated this pattern of results among a sample of chronic pain clinic attendees (McParland, Knussen, Serpell, & Eccleston, 2014). However, it did find the adaptive relationship between the personal just world belief and pain could be at least partially explained by activity engagement, such that those with a strong personal just world belief reported less pain when they continued to pursue desired life activities despite pain (McParland et al., 2014). These findings suggest that maintaining a belief in a just world can be adaptive in the experience of distress and pain. Alternatively, the absence of a belief in justice or the presence of a weak just world belief can be detrimental to pain.

Currently, little is known about the mechanisms through which the belief in a just world, particularly the personal just world belief, exerts its influence in the chronic pain experience. Recently, pain catastrophizing, an exaggerated negative orientation towards pain that is consistently associated with adverse physical and psychological pain outcomes, has been proposed as a route through which justice-related beliefs are related to long-term pain (Quartana, Campbell, & Edwards, 2009; Sullivan, 2012). Research has found it to be negatively correlated with perceived injustice, defined as severity/irreparability of loss, and blame/unfairness in relation to injury (Scott & Sullivan, 2012; Sullivan et al., 2008). It is also theorized to mediate the effects of perceived injustice on adverse pain outcomes via rumination over suffering and loss (Sullivan, Yakobov, Scott, & Tait, 2014). However, the role of pain catastrophizing as a mediator of the relationship between the personal belief in a just world and pain outcomes has not yet been explicitly examined.

The present study undertook a secondary data analysis to investigate pain catastrophizing as a mediator of the relationship between the personal belief in a just world and chronic pain outcomes. We acknowledge that the belief in a just world and perceived injustice are theoretically distinct from each other. However, considering the findings and suggestions of previous research, i.e. that pain catastrophizing is positively associated with adverse pain outcomes and perceived injustice, and that the personal belief in a just world is associated with less pain, disability and distress, we investigated two hypotheses. These hypotheses were driven by the assumptions that the personal belief in a just world is relatively stable and that it functions as a personal resource in everyday life and in stressful situations, while catastrophizing can be construed as a form of secondary appraisal that is associated with specific ways of coping with a stressful situation (Knussen & McParland, 2009). We predicted that a stronger personal belief in a just world would result in lower levels of catastrophizing and that the relationship between the belief and pain outcomes would be mediated by catastrophizing. More specifically, the first hypothesis was that pain catastrophizing would be negatively correlated with the personal belief in a just world. The second hypothesis was that catastrophizing would mediate the relationships found in the primary analysis between the personal just world belief and pain intensity, General Health Questionnaire (GHQ) scores, and pain-related disability (see McParland & Knussen, 2010). Since pain is experienced in a complex social and cultural milieu where justice issues are likely to be salient, understanding the role of a strong belief in justice as a potential resource in supporting attempts to cope with pain is an important undertaking that may help to inform theoretical and intervention development.

## Methods

### Participants

Participants were recruited from 11 arthritis and 4 fibromyalgia support groups. Questionnaire packs containing measures of physical and psychological functioning were distributed at the group meetings. Completed packs were returned either via post or through collection at a group meeting. The sample consisted of 95 participants. The largest proportion of participants reported they experienced arthritis (*n* = 43, 45 %); 15 participants (16 %) reported fibromyalgia, and the remaining participants reported back pain (*n* = 10, 10.5 %), joint or muscular pain (*n* = 15, 16 %) or undefined pain (*n* = 12, 13 %). The mean age of the sample was 66.23 years (SD = 11.44, range = 43 to 93 years), and the mean pain duration was 16.21 years (SD = 14.66, range = 6 months to 68 years). Most participants (*n* = 87, 97.6 %) were female.

### Measures

#### Pain Intensity

The Chronic Pain Grade (Von Korff, Ormel, Keefe, & Dworkin, 1992) was used to measure current, average and worst pain intensities over the previous 6 months. Items are scored on a progressive 10-point scale and are summed to provide separate pain intensity scores. Overall pain intensity was recorded as the average of current, average and worst self-reported pain. This measure has achieved adequate internal reliability among individuals experiencing chronic pain (McParland & Knussen, 2010; Smith et al., 1997). It has also achieved adequate validity (Elliot, Smith, Cairns Smith, & Chambers, 2000; Smith et al., 1997).

#### Self-Rated Disability

We measured self-rated disability using the Roland-Morris Disability Scale (Roland & Morris, 1983). This scale is a 24-item measure containing response options “Yes” and “No” to statements about disability in daily activities experienced in the past 2 weeks. The response options are scored such that a score of 1 is allocated to a “Yes” response and a score of 0 is awarded to a “No” response. The total score is obtained by summing the number of “Yes” responses. A higher score indicates greater self-reported disability. The original scale was developed to measure back pain. We modified the scale slightly by removing the word “back” and replacing it with “pain” to make it applicable to all pain sufferers. The measure has demonstrated satisfactory reliability and validity (McParland et al., 2014; Roland & Fairbank, 2000).

#### Personal Belief in a Just World

The personal belief in a just world was measured using the Personal Belief in a Just World Scale (Dalbert, 1999). This scale is a seven-item measure yielding a six-point Likert scale response range in which the level of agreement with items about the justness of events in one’s own life is indicated; for example, “I believe that, by and large, I deserve what happens to me.” Scores on each of the seven items are summed to provide a total personal belief in a just world score. Higher scores indicate a stronger endorsement of the belief. The scale has achieved adequate internal validity in chronic pain samples (McParland & Knussen, 2010; McParland, Knussen, Lawrie, & Brodie, 2013; McParland et al., 2014) and is a valid measure (Dalbert, 1999, 2000).

#### Distress

The level of psychological distress was measured using the 28-item General Health Questionnaire (GHQ) (Goldberg & Hillier, 1979). The scale contains four subscales that measure somatic symptoms, anxiety and insomnia, social dysfunction and severe depression experienced in the previous few weeks. Responses were scored on a 0–3 scale, with higher scores indicating a poorer outcome. The items of each subscale were summed to provide a total psychological distress score. The GHQ has been used reliably in our research (McParland & Knussen, 2010) and is a valid measure (Goldberg & Hillier, 1979; Wernecke, Goldberg, Yalcin, & Ustun, 2000).

#### Catastrophizing

The six-item catastrophizing subscale of the Coping Strategies Questionnaire (Rosenstiel & Keefe, 1983) was used to measure pain catastrophizing. The subscale measures elements of helplessness and pessimism in relation to one’s ability to deal with the pain experience. Individuals rate on a 0 (Never do that) to 6 (Always do that) scale the extent to which they experience the thought or feeling described by each item when they are in pain. The scale has strong psychometric properties (Robinson et al., 1997), internal consistency of the subscale is within an acceptable range (Rosenstiel & Keefe, 1983) and test-retest reliability is adequate (Main & Waddell, 1991).

## Data Analysis

Data analysis was conducted using IBM SPSS Statistics version 21. Missing responses were examined, and data were imputed using the multiple imputation command if fewer than 25 % of the items within any one scale had been omitted and if the participant had omitted fewer than 50 % of all items. Relationships between continuous variables were examined using Pearson’s correlations. The criteria suggested by Kraemer, Kiernan, Essex and Kupfer (2008) were used to examine mediation: just world beliefs were assumed to precede the mediator, catastrophizing; a significant relationship was required between just world beliefs and catastrophizing and between both and the outcome variables; and the interaction term was examined in the models. Hierarchical multiple regression analyses were conducted to examine mediation, and all variables were centred prior to inclusion. The interaction term was computed by multiplying the two centred variables. Covariates and the centred variables were entered prior to the interaction term. Sobel tests were conducted to determine whether significant mediation could be demonstrated.

## Results

Table 1 shows the descriptive statistics for the sample and the correlations among the study variables. Some of these results are presented in our previous papers (Knussen & McParland, 2009; McParland & Knussen, 2010) and so will not be discussed here. However, it is relevant to note that the samples were mostly older with long-standing pain and a moderate self-reported level of pain. The key findings for the current analysis are that personal just world belief scores were negatively associated with pain catastrophizing and both were significantly related to all three outcome variables (pain intensity, self-reported disability and psychological distress).

**Table 1:** Characteristics and descriptive statistics for the sample

	Mean (SD)	Range	*α*	Age	Duration	Pain intensity	RM Disability	GHQ	Personal BJW
Age (years)	66.23 (11.44)	43–93	–						
Duration of pain (years)	16.21 (14.66)	0.6–68	–	.13					
Pain intensity	6.84 (2.12)	0–10	0.88	−.20	−.21				
RM Disability	14.51 (5.96)	0–24	0.91	−.23*	−.26*	.64***			
GHQ	24.62 (11.75)	6–53	0.94	−.25*	−.11	.39***	.51***		
Personal BJW	25.72 (6.06)	13–42	0.70	−.37***	−.11	−.25*	.21*	−.45***	
Catastrophizing	1.92 (1.50)	0–6	0.87	−.22*	−.18	.48***	.61***	.55***	−.29**

*Personal BJW* Personal Belief in a Just World, *RM Disability* Roland-Morris Disability, *GHQ* General Health Questionnaire

*p < .05; **p < .01; ***p < .001

We expected catastrophizing to mediate the relationships between the personal just world belief and the outcome variables. Given the correlations among the study variables, the main conditions for mediation analysis were met (Kraemer et al., 2008): the association between the potential mediator (catastrophizing) and the target variable (personal just world belief) was significant, and it could be assumed that the target variable had temporal precedence over the mediator (in that the establishment and degree of belief in a just world was assumed to precede pain catastrophizing). The results are shown in Table 2. The interaction term (Personal Just World Beliefs × Catastrophizing) was not significant in any of the analyses and was omitted to aid interpretation. There was no indication that catastrophizing mediated the relationship between the personal just world belief and psychological distress (GHQ): the relationship remained significant when catastrophizing entered the equation. With both disability and pain intensity, the relationships with the personal just world belief lost significance when catastrophizing entered the equation, suggesting mediation. Sobel tests (Preacher & Leonardelli, 2001) were conducted, and both suggested significant mediation of the relationships between catastrophizing and both disability and pain by the personal just world belief: for disability, *z* = −2.51, SE = 0.00, *p* = .012; and for pain intensity, *z* = −2.39, SE = 0.02, *p* = .017.

**Table 2:** Test of mediation

		GHQ						RM Disability						Pain Intensity					
		*Β*	95 % CI	*β*	*t*	*p*	Δ*R* ^2^	*Β*	95 % CI	*β*	*t*	*p*	Δ*R* ^2^	*Β*	95 % CI	*β*	*t*	*p*	Δ*R* ^2^
Step 1	Personal BJW	−0.88	−1.24 to −0.52	−0.46	−4.83	<.001	.21	−0.01	−0.02 to 0.00	−0.22	−2.08	.040	.05	−0.09	−0.16 to −0.02	−0.26	−2.46	.016	.07
Step 2	Personal BJW	−0.63	−0.96 to −0.30	−0.33	−3.77	<.001		−0.00	−0.01 to 0.01	−0.06	−0.61	.541		−0.04	−0.11 to 0.02	−0.13	−1.31	.195	
	Catastrophizing	3.57	2.22 to 4.92	0.45	5.24	<.001	.19	0.10	0.07 to 0.13	0.59	6.58	<.001	.32	0.61	0.34 to 0.88	0.43	4.43	<.001	.17

*Personal BJW* Personal Belief in a Just World, *RM Disability* Roland Morris Disability, *GHQ* General Health Questionnaire

## Discussion

The present study conducted a secondary data analysis to investigate whether pain catastrophizing mediated the relationships that the personal belief in a just world held with pain intensity, disability and psychological distress among a sample of chronic pain support group attendees. As reported in our previous research, the personal belief in a just world was negatively correlated with pain, disability and psychological distress (McParland & Knussen, 2010). Pain catastrophizing was positively associated with these variables (Knussen & McParland, 2009). These findings are consistent with previous research which suggests that the personal just world belief is important for wellbeing (see Dalbert, 2009). The findings also support research that has found pain catastrophizing to be a consistent predictor of pain, disability and psychological distress among individuals with chronic pain in general (Severeijns, Vlaeyen, Van Den Hout, & Weber, 2001) and a critical factor in explaining the experience of pain in rheumatic diseases and fibromyalgia (Edwards, Bingham, Bathon, & Haythornthwaite, 2006; Edwards et al., 2010; Edwards, Cahalan, Mensing, Smith, & Haythornthwaite, 2011). In a new finding, the personal belief in a just world was negatively associated with pain catastrophizing. Given that pain catastrophizing has been positively associated with perceived injustice (Scott & Sullivan, 2012; Sullivan et al., 2008), this inverse association between both constructs is unsurprising.

Mediational analyses found that pain catastrophizing helped to explain the relationship that the personal belief in a just world held with pain and disability, but not psychological distress. This latter finding is consistent with previous research indicating that the belief in a just world for the self is uniquely related to psychological wellbeing (Sutton & Douglas, 2005). However, it is possible that other factors mediated the relationship between the personal belief in a just world and psychological distress. In particular, research has found that strong just world believers are likely to make internal rather than external attributions for their negative outcomes, leading to fewer perceptions of unfairness, which can result in reduced negative affect (Hafer & Correy, 1999). Thus, attributions may be relevant variables to consider in future research investigating the relationship between the belief in a just world and emotional outcomes in pain.

One tentative explanation as to why pain catastrophizing mediated the effects of pain and disability relates to our previous theorizing of the pain catastrophizing as an aspect of secondary appraisal in the transactional model of stress and coping (Folkman, 1984; Lazarus, 1990) (see Knussen & McParland, 2009). In this interpretation, pain is considered to be an overwhelming experience and little or nothing can be done for it. Applied to our study results, it is possible that having a weak personal belief in a just world leaves an individual vulnerable to negative thoughts, in this case helplessness and pessimism which can increase pain and disability. In support of this idea, in a related area of research, the belief in an unjust world has been associated with maladaptive cognitions including neuroticism, defensive coping and perceived future risk (Lench & Chang, 2007). Additionally, the helplessness and pessimism components of catastrophizing measured in this study are consistently associated with negative physical and psychological functioning in research (e.g. Brenes, Rapp, Rejeski, & Miller, 2002; Muller, 2011; Ramirez-Maestre, Esteve, & Lopez, 2012; Samwel, Evers, Crul, & Kraaimaat, 2006).

In an alternative explanation, we speculate that a strong personal belief in a just world might be associated with less pain, disability and distress because it protects against thoughts of helplessness and pessimism. This interpretation is consistent with that of previous research that has found pain catastrophizing to be a mediator between positive traits and pain outcomes. In the first study in this area, Hood, Pulvers, Carrillo, Merchant, and Thomas (2012) found all three rumination, magnification and helplessness components of pain catastrophizing (Sullivan, Bishop, & Pivik, 1995) independently mediated the relationships between hope, optimism and pain among a sample of healthy individuals exposed to the cold pressor task. Additionally, Goodin et al. (2013) found that pain catastrophizing mediated the relationship between optimism and temporal summation among a sample of osteoarthritis sufferers. Pulvers and Hood (2013) proposed a model suggesting that low levels of pain catastrophizing explain the link between positive traits and pain perception. However, as acknowledged by the authors, research is needed to identify whether positive traits reduce pain catastrophizing or whether low pain catastrophizing increases positive traits to provide direction for intervention development and reduce pain. To the extent that the personal belief in a just world can be viewed as a positive variable in the pain experience, it will be worthwhile to investigate whether a strong personal just world belief reduces the potential for negative thoughts or whether low pain catastrophizing increases the strength of this belief.

Regardless of the direction of findings, future research in this area that considers other conceptualizations of catastrophizing, in terms of magnification and rumination (Sullivan et al., 1995), is warranted. We speculate this research may, for instance, reveal low rumination over one’s situation as a strategy to preserve the personal just world belief when faced with personal injustice in pain (Dalbert, 1997). Future research should consider the severity of injustice in this investigation as there is evidence that, contrary to popular wisdom, the belief in a just world is most likely to be threatened by milder rather than severe injustice (Corey, Troisi, & Nicksa, 2015). In the latter circumstances, a strong belief in a just world is needed as a personal resource to help someone to cope with the injustice (Corey et al., 2015).

The results of the present study suggest that interventions to reduce pain catastrophizing would be helpful among those with a weak belief in a just world. Research suggests that cognitive behavioural therapy is effective in addressing pain catastrophizing (Stonerock, 2012). Cognitive behavioural interventions that include graded exposure, graded activity increase, cognitive reappraisal and goal setting have been shown to reduce catastrophizing and associated disability. However, relatively little is known about interventions for promoting justice-related beliefs. Developing guidelines that promote fairness in different contexts may be a relevant future direction to attenuate negative responses to unfavourable outcomes. For example, Greenberg (2006) found employee health benefits of using interactional justice techniques to train their supervisors in the provision of respectful treatment, emotional support and effective communication to enable them to help their employees to accept an unfavourable outcome (a reduction in salary). Such primary prevention techniques provide an important starting point for considering justice interventions to promote improved wellbeing among individuals who live with chronic pain. For example, the implementation of such techniques may help chronic pain patients to cope with unmet expectations related to their medical treatment, something that is a source of perceived injustice (McParland et al. 2011a, McParland, Hezseltine, Stenner, Serpell, & Eccleston, 2011b).

There are many shortcomings of the research, which are reported in our previous paper (McParland & Knussen, 2010). Specifically, since most of the participants were female, and older, the results cannot be generalized to other pain populations. Research suggests that the meaning of the belief in a just world can vary by age, with older individuals using the belief to interpret their lives in a meaningful way and to avoid rumination over negative aspects of their lives (Dalbert, 2009). Additionally, males and females may believe in a just world for different reasons, and this belief can be linked to different outcomes for males and females (Benson, 1992). Thus, further research is needed to investigate the wellbeing function of the personal belief in a just world among younger populations living with chronic pain, particularly males. Secondly, the cross-sectional, correlational nature of the study precludes suggestions about the causal relationship among the study variables. Finally, the study is dependent on data collected using self-report measures.

Despite these limitations, this study has shown an association between adaptive and maladaptive cognitions in the experience of pain through flagging pain catastrophizing as a mediator in the relationship of the personal just world belief with pain and disability. The findings add to what is already known about how the personal just world belief exerts its effects on pain (McParland et al., 2014). The results suggest that, although in need of closer investigation, the effects of the personal belief in a just world on pain outcomes may occur via the action of low pain catastrophizing. Future research is needed to prospectively and/or experimentally test the causal pathways suggested here and to develop interventions targeting both the belief in a just world and pain catastrophizing.
